# Multiscale model to investigate the effect of graphene on the fracture characteristics of graphene/polymer nanocomposites

**DOI:** 10.1186/1556-276X-7-595

**Published:** 2012-10-26

**Authors:** Avinash Parashar, Pierre Mertiny

**Affiliations:** 1 University of Alberta, 4-9 Mechanical Engineering Building, Edmonton, Alberta, T6G 2G8, Canada

**Keywords:** Nanocomposite, Polymers, Fracture, Finite element analysis

## Abstract

In this theoretical research work, the fracture characteristics of graphene-modified polymer nanocomposites were studied. A three-dimensional representative volume element-based multiscale model was developed in a finite element environment. Graphene sheets were modeled in an atomistic state, whereas the polymer matrix was modeled as a continuum. Van der Waals interactions between the matrix and graphene sheets were simulated employing truss elements. Fracture characteristics of graphene/polymer nanocomposites were investigated in conjunction with the virtual crack closure technique. The results demonstrate that fracture characteristics in terms of the strain energy release rate were affected for a crack lying in a polymer reinforced with graphene. A shielding effect from the crack driving forces is considered to be the reason for enhanced fracture resistance in graphene-modified polymer nanocomposites.

## Background

Nanocomposites composed of nanofiller reinforcement and a polymer matrix are currently subject to intense research due to possible improvements in physical, mechanical, and/or electrical properties compared to neat polymer. Graphene is emerging as a potential candidate for nanoscale reinforcement of polymer nanocomposites. Stankovich et al. [1] introduced a novel technique for mass-producing graphene at comparatively low cost, which provides the opportunity of using graphene for a variety of conventional purposes and applications, such as improving the characteristics of adhesively bonded joints.

In their experimental works, Rafiee et al. [2,3] observed increased fracture toughness of graphene-modified epoxy nanocomposites. They reported that the addition of graphene sheets into the epoxy matrix resulted in a distinct increase in fracture toughness, i.e., fracture toughness of epoxy was increased by up to 65% with an inclusion of 0.125% weight fraction of graphene [2]. It was also reported in their experimental work that a uniform distribution of graphene sheets in an epoxy matrix remains a challenging undertaking, which currently limits the full understanding of the mechanisms behind the property improvements. Hence, modeling is considered a viable alternative to explore the effects of nanofiller dispersion on the fracture properties of polymer nanocomposites.

The number of theoretical/numerical works published on graphene-based nanocomposites has so far been limited. Existing works in this field are, for example, the molecular dynamics-based simulation techniques employed by Awasthi et al. [4], who studied the load transfer mechanisms between polyethylene and a graphene sheet. Cho et al. [5] employed a Mori-Tanaka approach to study the elastic constants of nanocomposites with randomly distributed graphene sheets. Most recently, Montazeri and Tabar [6] developed a multiscale finite element model to study the elastic constants of a graphene-based polymer nanocomposite. Parashar and Mertiny [7] also proposed a multiscale model using finite elements to characterize the buckling phenomenon in graphene/polymer nanocomposites.

To the present authors' knowledge, no theoretical studies are available in the technical literature on the fracture behavior of graphene/polymer nanocomposites. In the present paper, an attempt has been made to develop a multiscale model to investigate the fracture characteristics of graphene-modified epoxy nanocomposites. The proposed multiscale modeling technique was developed in the ANSYS (version 13) finite element software environment (ANSYS Inc., Canonsburg, PA, USA) in conjunction with the virtual crack closure technique (VCCT). A multiscale approach employing the VCCT provides an efficient numerical analysis scheme in terms of the involved degrees of freedom. As a consequence, the analysis can be performed with widely available computational systems.

## Methods

In the present work, considering the polymer matrix and graphene nanofiller as a continuum and atomistic phase, respectively, a multiscale model in conjunction with finite element analysis was developed. The bond interaction between carbon atoms in graphene was simulated with the help of beam elements. In the current finite element simulation, the modified Morse potential was employed to model the bonded interaction between C-C bonds. The Morse potential has already been applied in a number of research works [8,9], where structures were subjected to large strain values.

According to the modified Morse potential, the potential energy of the isolated graphene sheet can also be expressed as the sum of the bond-stretching component (*E*_S_) and the angle-bending component (*E*_B_) as given in Equations 1 to 3. The variables and parameters required for Equations 1 to 3 can be found in [10] (see Table 1).

(1) $$E=E_{S}+E_{B}$$

(2) $$E_{\text{S}}=D_{\text{e}}\left\{{\left[1-{\text{exp}}^{-\beta \left(r-r_{\text{o}}\right)}\right]}^{2}-1\right\}$$

(3) $$E_{\text{B}}=\frac{1}{2}k_{\theta }{\left(\theta -\theta_{\text{o}}\right)}^{2}\left[1+k_{\text{sextic}\;}{\left(\theta -\theta_{\text{o}}\right)}^{4}\right]$$

**Table 1:** Modified Morse potential variables and parameters

**Parameter**	**Value**	**Description**
*r* (m)	-	Length of C-C bond
*r*_o_ (m)	0.1421 × 10^−9^	Equilibrium C-C bond distance in graphene
*ε*	(*r* − *r*_o_) / *r*_o_	Strain in C-C bond
*D*_e_ (Nm)	6.03105 × 10^−19^	Dissociation energy
*β* (m^−1^)	2.625 × 10^10^	Constant controlling the ‘width’ of the potential
*θ* (rad)	-	Current angle of the adjacent bond
*θ*_o_ (rad)	2.094	Initial angle of the adjacent bond
*k*_*θ*_ (Nm/rad^2^)	0.9 × 10^−18^	Force constant for bond bending
*k*_sextic_ (rad^−4^)	0.754	Constant in bending term of potential

In the current research paper, the bond-stretching component dominates the overall bond energy, and therefore, the angle-bending component was neglected in the simulation. The interatomic force acting between the two C-C atoms can be explored by differentiating Equation 2 to give

(4) $$F=2\beta D_{\text{e}}\left(1-{\text{exp}}^{-\beta \varepsilon r_{\text{o}}}\right){\text{exp}}^{-\beta \varepsilon r_{\text{o}}}$$

**Figure 1:**
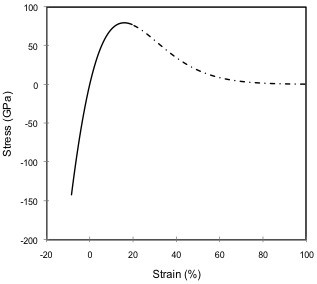
Non-linear stress–strain relation for a C-C bond developed from the Morse potential.

Elastic beam elements ‘BEAM4’ were employed in this work to simulate C-C bond-stretching. The cross-sectional area of each beam element was estimated to be 0.09079 nm^2^ (the diameter of each beam element was considered equal to the thickness of the graphene sheet, that is, 0.34 nm), which was further employed in conjunction with Equation 4 to model the non-linear stress–strain behavior of C-C bonds (plotted in Figure 1). From the initial slope of data plotted in Figure 1, the initial stiffness of C-C bonds was assigned as 1.33 TPa.

**Figure 2:**
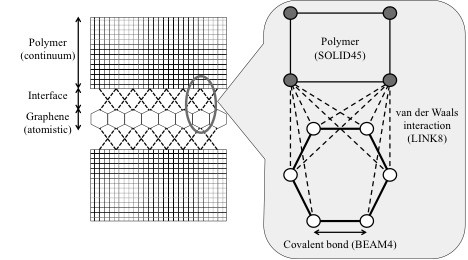
Schematic diagram of a multiscale model.

**Figure 3:**
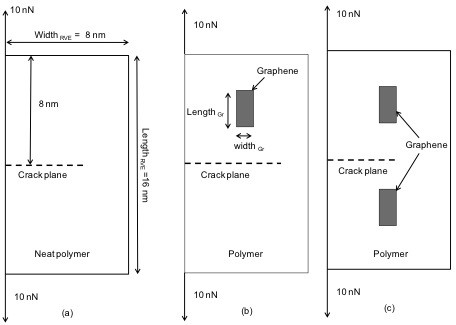
**Schematic of model configurations:** (**a**) neat polymer with crack, (**b**) single graphene/polymer nanocomposite, and (**c**) twin graphene/polymer nanocomposite.

The continuum phase (polymer) of the multiscale model as shown in Figure 2 was meshed with a three-dimensional continuum element (SOLID45). The continuum phase was modeled with a Young's modulus of *E* = 3.4 GPa and with a value of 0.42 for Poisson's ratio. In general, the continuum mesh size in such multiscale models was kept to the same size as the individual cell of the carbon structure [6], but in this paper, a more specific approach was employed. To formulate the interphase between the graphene and polymer, meshing of the continuum phase in the interphase region was done with a specific mapped mesh density. In the research work published in [11], epoxy was modeled as polymer chains, where the spacing between the chains was kept at 0.3816 nm, which is the equilibrium spacing with respect to van der Waals forces. According to the above justification, the element (SOLID45) thickness and width was kept at 0.3816 nm, which is the same as the equilibrium distance due to van der Waals interactions. The element size along the length of the representative volume element (RVE; see Figure 3) was kept at 0.125 nm. The specified element density was kept in the near vicinity of the graphene/polymer interphase up to a distance of 0.85 nm from the graphene edge, whereas different mesh specifications were employed to mesh the region containing the crack plane.

The most significant part of the proposed multiscale model is the interphase region between the atomistic graphene model and the continuum polymer representation (see Figure 2). A number of approaches have been considered to account for the interfacial properties and thickness. These depend on the type of bonding, i.e., functionalized or non-functionalized bonding, as well as on the load transfer mechanism through the interface. Hence, the interfacial properties have not unambiguously been defined yet. Hu et al. [12] in their work on polystyrene and carbon nanotubes (CNT) considered van der Waals interactions to be responsible for maintaining interfacial strength. They assumed 0.2851 to 0.5445 nm as the interface thickness. Meguid et al. [11] simulated the interaction between the CNT and polymer chains with an interfacial thickness of 0.3816 nm. In the present work, the interfacial thickness was set to 0.172 nm, which is consistent with that in the numerical model proposed by Li and Chou [13].

For simulating the van der Waals interactions at the graphene/polymer interphase, a truss model (LINK8) was employed as illustrated in Figure 2. The activation of a truss element is determined by the distance between an atom in the graphene sheet and a node in the continuum state model, that is, a truss element is activated when the distance between an atom/node in the graphene and a node in the continuum mesh for the polymer material is less than or equal to 0.65 nm.

Mechanical properties for the truss elements were determined in a similar manner to that presented in the research work by Odegard et al. [14], namely the Lennard-Jones (LJ) ‘6-12’ potential given in Equation 5 was considered for simulating van der Waals interactions:

(5) $$U\left(r\right)=4\phi \left[{\left(\frac{\phi }{r}\right)}^{12}-{\left(\frac{\phi }{r}\right)}^{6}\right]\text{,}$$

where *r* is the distance between two atoms, *ϕ* is the hard sphere radius (*ϕ =* 0.34 m), and *ϕ* is the potential well depth (*ϕ* = 0.0556 kcal/mol). The mechanical properties for the truss elements were obtained by equating the LJ ‘6-12’ potential with the structural strain energy of truss elements. The resulting expression is provided in Equation 6 and was used for assigning material stiffness values to each truss element with a cross-sectional area of 0.0907 nm^2^:

(6) $$E\left(r\right)=\frac{8\phi R_{\text{Eq}}}{A{\left(r-R_{\text{Eq}}\right)}^{2}}\left[{\left(\frac{\phi }{r}\right)}^{12}-{\left(\frac{\phi }{r}\right)}^{6}\right]$$

**Figure 4:**
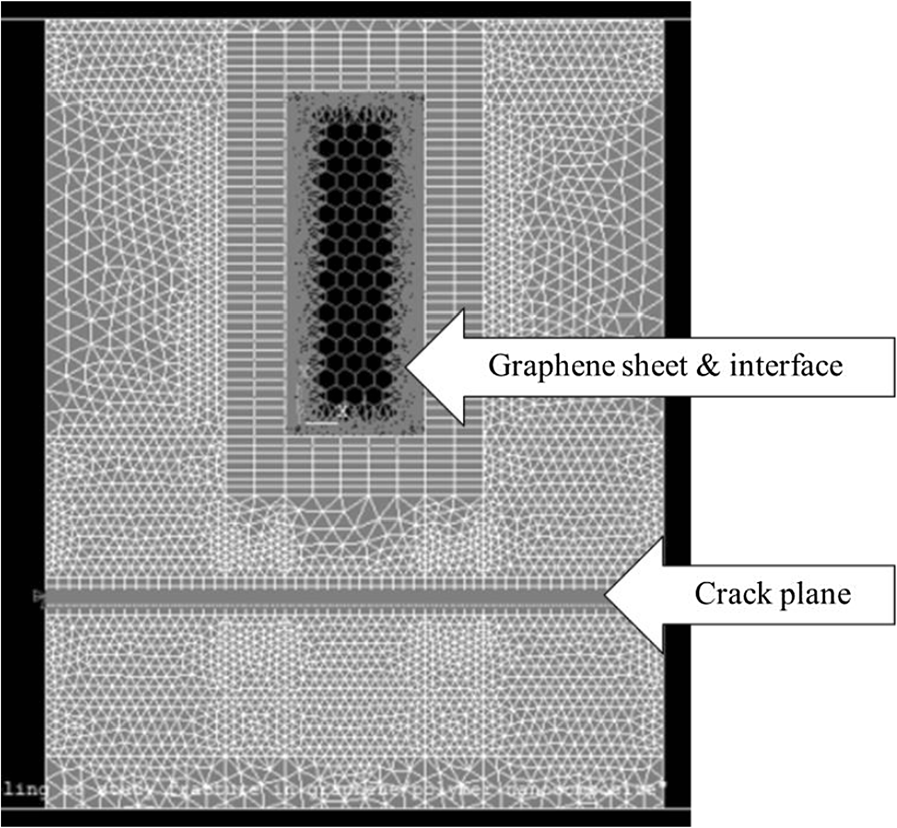
Finite element structure of multifunctional model.

Figure 4 portrays the actual finite element structure for a developed multifunctional model configuration showing the respective elements for the continuum polymer phase (SOLID45), the atomistic graphene structure (BEAM4), and their interface (LINK8). The thickness of graphene sheets and continuum polymer phase was kept at 0.344 and 0.3816 nm, respectively, for all model configurations investigated in this study. Nodes with coupled degrees of freedom are also depicted in Figure 4. These nodes constitute the crack plane, that is, crack propagation was simulated by the subsequent release of node constraints. The proposed RVE simulated the crack growth under plane stress conditions as the thickness of the RVE under consideration is comparably smaller than the other dimensions.

**Figure 5:**
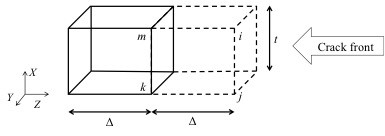
Schematic of crack front in eight-node three-dimensional elements.

In this work, the virtual crack closure technique was employed in conjunction with a multiscale model to study the fracture characteristics of graphene/polymer nanocomposites under opening-mode crack propagation. The strain energy release rate (SERR) mode I (*G*_I_) was considered as the characterizing parameter in this paper. The mathematical formulation based on Figure 5 is provided in Equation 7 for estimating *G*_I_[15]:

(7) $$G_{I}=\frac{1}{2\text{t}\Delta }\left\{F_{\mathrm{iy}}\left(v_{m}-v_{m'}\right)+F_{\mathrm{jy}}\left(v_{k}-v_{k'}\right)\right\}$$

## Results and discussion

The proposed RVE was developed in the ANSYS finite element environment as discussed in the preceding section. Schematics for the different model configurations along with boundary conditions, an applied opening load of 10 nN, and coordinates are shown in Figure 3. As stated earlier, crack propagation was modeled in isotropic epoxy as the polymer. The thickness of the RVE was small compared with the other dimensions of the RVE, which caused the analysis to fall under plane stress conditions. Calculations for the graphene volume fraction involved in the RVE were performed according to Equation 8:

(8) $$VF=\frac{length_{\mathrm{Gr}}\times width_{\mathrm{Gr}}\times thickness_{\mathrm{Gr}}}{length_{\mathrm{RVE}}\times width_{\mathrm{RVE}}\times thickness_{\mathrm{Gr}}}$$

Here, VF represents the graphene volume fraction, and the subscripts Gr and RVE designate the dimensions of the graphene sheet and the representative volume element, respectively, as illustrated in Figure 3. The graphene considered in the multiscale models had 19 cells fixed along the length of the RVE, whereas a different number of cells was employed along the width of the RVE according to the graphene volume fraction. Three cells correspond to a volume fraction of 2.125%, and hence, 6 and 12 cells were used to model volume fractions of 4.25% and 8.5%, respectively.

**Figure 6:**
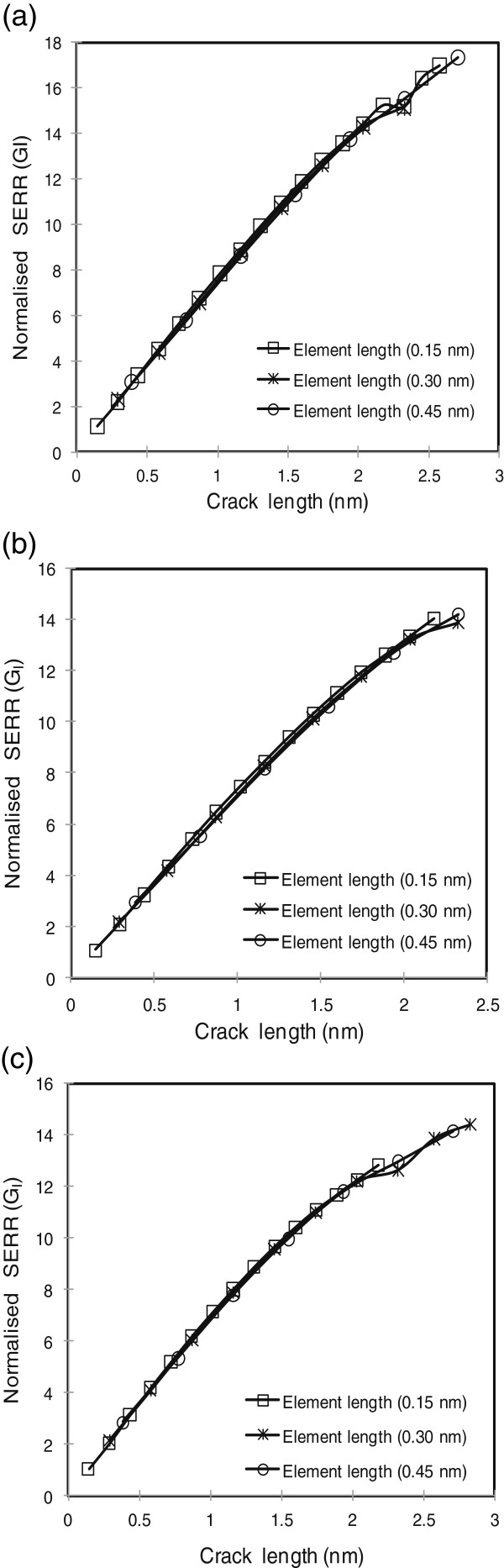
**Convergence results.** (**a**) Neat epoxy model. (**b**) Single graphene sheet configuration. (**c**) Twin graphene sheet configuration.

SERR *G*_I_ values obtained for the crack shown in Figure 3a,b,c were tested for convergence. Convergence results plotted in Figure 6a,b,c correspond to the model developed for pure epoxy (Figure 3a), the single graphene sheet with a size of 4.1209 × 1.47 nm (Figure 3b), and two graphene sheets also with a length and width of 4.1209 and 1.47 nm (Figure 3c), respectively. It can be concluded from the plots in Figure 6 that SERR *G*_I_ values converged to a finite value even for a coarse mesh size. In this study, an element length of 0.3 nm was considered for modeling the crack tip coordinates. The multiscale model was employed to study the effect of shielding, volume fraction, dispersion, and aspect ratio of graphene on the fracture toughness of graphene/polymer nanocomposites.

### Significance of graphene volume fraction

Initially, SERR values were estimated for a crack propagating in neat polymer with boundary conditions as shown in Figure 3a. Resulting SERR values were considered as reference values (*G*_IR_) for subsequent investigations.

**Figure 7:**
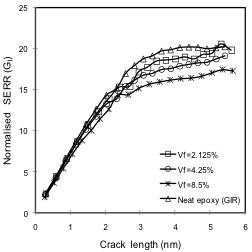
**Effect of graphene volume fraction on SERR *G*_I_.**

In the first part of this study, the effect of graphene volume fraction on *G*_I_ cohesive crack growth was investigated, considering boundary conditions as illustrated in Figure 3b. Results obtained from the simulations are shown in Figure 7. It can be inferred from the data plotted in this figure that crack growth in terms of SERR *G*_I_ was reduced with the increase in graphene volume fraction. In comparison to the neat epoxy model (i.e., aforementioned *G*_IR_ values), an improvement of up to 6% in fracture toughness (when the crack passes in the vicinity of the graphene sheet) was observed for a graphene volume fraction of 2.125%, which increased to 18% for a volume fraction of 8.5%. Note that in the current modeling approach, an increase in graphene volume fraction is associated with a reduction of the graphene sheet aspect ratio, which is defined as the length to width ratio. The influence of graphene aspect ratio will further be discussed in the following sections.

Observed improvements in fracture toughness of graphene nanocomposites were attributed to the change in stress distribution in the (continuum) polymer epoxy phase due to the graphene inclusion in the vicinity of the crack. It is postulated that graphene with its space frame structure and high stiffness bears most of the applied load and shields the crack tip from opening loads or crack-driving forces, whereas higher SERR *G*_1R_ values in neat epoxy can be attributed to the absence of this shielding effect.

### Shielding effects in graphene/polymer nanocomposites

**Figure 8:**
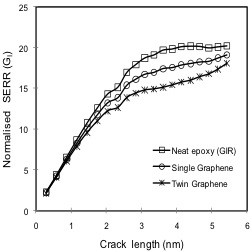
**Effect of graphene dispersion on SERR *G*_I_.**

To further investigate the influence of graphene on fracture characteristics and the aforementioned shielding effect of graphene in graphene/polymer nanocomposites, modeling was performed with all three models as described in Figure 3. Again, the SERR *G*_IR_ values obtained according to Figure 3a were compared with *G*_I_ values employing the conditions defined in Figure 3b,c. Corresponding results are plotted in Figure 8. Legend entries of ‘Neat epoxy’, ‘Single graphene,’ and ‘Twin graphene’ in this figure correspond to SERR values obtained from models defined in Figure 3a,b,c, respectively. The graphene sheets considered in the RVE for the ‘Single graphene’ and ‘Twin graphene’ cases had a fixed aspect ratio of 2.8, i.e., length and width were 4.1209 and 1.47 nm, respectively. The graphene volume fractions were correspondingly 4.25% and 8.5%.

It can be inferred from the SERR values plotted in Figure 8 that a significant improvement in fracture characteristics was obtained for the nanocomposite reinforced with a graphene sheet on both sides of the crack plane. For this configuration, *G*_I_ values were reduced by up to 24% (for crack propagation near the graphene sheet) compared with the *G*_IR_ values. Modeling results therefore indicate that the crack tip shielding effect from crack driving forces is more pronounced when graphene is present next to the crack on both sides of the crack plane. It shall be mentioned at this point that an improvement in SERR also occurs when a ‘Single graphene’ model with a graphene volume fraction identical to that of the ‘Twin graphene’ case is considered, which is shown in the subsequent section.

### Graphene dispersion and dimensional effects

Earlier experimental works [2,3] showed that graphene/polymer nanocomposites have enhanced fracture toughness compared to neat polymer. Due to challenges associated with the mixing of graphene in polymer, none of the studies have thus far been devoted to evaluating the effect of graphene dispersion and its aspect ratio on nanocomposite fracture toughness. Experimental results mostly dealt with graphene volume fraction and its impact on fracture toughness. This section of numerical analysis was conducted to study the effect of graphene aspect ratio and its distribution in the polymer matrix on fracture toughness of the developed nanocomposite.

**Figure 9:**
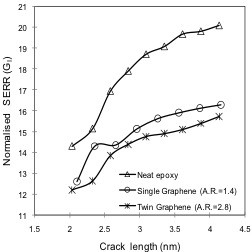
**Effect of graphene A.R. and dispersion on SERR *G*_I_ for graphene volume fraction of 8.5%.**

**Figure 10:**
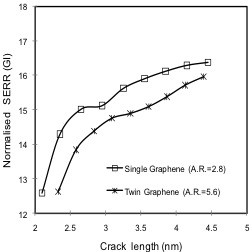
**Effect of graphene A.R. and dispersion on SERR *G*_I_ for graphene volume fraction of 4.25%.**

The multiscale models as defined in the schematics in Figure 3b,c were again used in this final part of the analysis. Results obtained from the model defined by Figure 3b, i.e., graphene with a low aspect ratio, are referred to as ‘Single graphene’ in Figures 9 and 10. A graphene nanocomposite with high aspect ratio and uniform dispersion was simulated using the model from Figure 3c. The length of the graphene sheets (length_Gr_) employed in the above models was kept constant at 4.1209 nm. The sheet width, however, was adjusted, that is, for the model with high aspect ratio and uniform dispersion (Figure 3c), half the width (width_Gr_) of the graphene sheet was employed compared to the model with low aspect ratio (Figure 3b). The analysis in this section was performed for two different cases, i.e., graphene volume fractions of 4.25% and 8.5%.

First, modeling was performed for a graphene volume fraction of 8.5%. Corresponding SERR values are plotted with respect to crack length in Figure 9, where legend entries of ‘Single graphene’ and ‘Twin graphene’ represent graphene with an aspect ratio (A.R.) of 1.4 and 2.8, respectively. In Figure 9, the maximum improvement in fracture toughness (crack passing the graphene sheet) compared to the neat polymer was approximately 18% for the ‘Single graphene’ case, whereas the improvement was about 24% for the ‘Twin graphene’ model, which is a difference of 6 percentage points. These results indicate that nanocomposite fracture toughness improves with increasing graphene aspect ratio as well as for nanofillers being uniformly distributed in the matrix.

To corroborate the above findings, modeling was performed next for a graphene volume fraction of 4.25%, and results are shown in Figure 10. Here, legend entries of ‘Single graphene’ and ‘Twin graphene’ correspond to graphene aspect ratios of 2.8 and 5.6, respectively. Figure 10 again shows a superior performance for the ‘Twin graphene’ configuration, yet improvements in SERR were lower in absolute terms than those for the previous case with a higher graphene volume fraction. Notably, for this lower graphene volume fraction as well as the higher graphene aspect ratios, the fracture toughness improvement for the ‘Twin graphene’ configuration compared to the ‘Single graphene’ model was greater, which is now 10 percentage points (compared to 6 percentage points in the previous analysis with a graphene volume fraction of 8.5%). Based on these results, it is postulated that the greatest enhancement of fracture toughness can be expected from a nanographene filler that is uniformly distributed in the polymer matrix and has high-aspect-ratio graphene sheets.

## Conclusions

A multiscale modeling technique in conjunction with a representative volume element approach was successfully employed in this investigation to study the fracture characteristics of graphene/polymer nanocomposites. Substantial improvements in the fracture characteristics of graphene/polymer nanocomposites were observed for higher volume fractions of graphene. It was concluded from this research that shielding of the crack tip from driving forces occurred in the vicinity of graphene sheets. In addition to graphene volume fraction, a uniform distribution of graphene in the polymer matrix as well as high aspect ratio of graphene sheets were found to enhance fracture toughness of graphene nanocomposites. Consequently, crack growth retardation can be achieved in graphene nanocomposites even without diverting or increasing the path of crack propagation.

## Competing interests

The authors declare that they have no competing interests.

## Authors’ contributions

Both authors made equally valuable contributions to this paper. Both authors read and approved the final manuscript.
